# Euthanasia: Manual versus Mechanical Cervical Dislocation for Broilers

**DOI:** 10.3390/ani9020047

**Published:** 2019-02-01

**Authors:** Leonie Jacobs, Dianna V. Bourassa, Caitlin E. Harris, R. Jeff Buhr

**Affiliations:** 1 Department of Animal and Poultry Sciences, Virginia Tech, Blacksburg, VA 24061, USA; 2 Department of Poultry Science, Auburn University, Auburn, AL 36849, USA; dvb0006@auburn.edu; 3 Department of Poultry Science, The University of Georgia, Athens, GA 30602, USA; ceh94@uga.edu; 4 USDA-ARS, US National Poultry Research Center, Athens, GA 30605, USA; jeff.buhr@ars.usda.gov

**Keywords:** culling, killing, poultry, euthanasia, cervical dislocation, reflexes, animal welfare, brain stem death

## Abstract

**Simple Summary:**

Poultry are euthanized for several reasons, most commonly because a bird is sick or injured and unable to eat or drink. Euthanasia can be a challenge to perform, especially when birds are heavy, like broiler chickens (produced for meat). Manual cervical dislocation (CD), or “breaking the neck”, is the most commonly applied method, but can be challenging. Therefore, using a tool (the mechanical method) such as the Koechner Euthanizing Device (KED) could be an alternative. Here, we aimed to compare CD with KED application for their impact on duration of induced reflexes and time to brain death. We assessed loss of brain stem reflexes, which indicate deep unconsciousness and/or brain stem death, and cessation of musculoskeletal movements. We applied both methods (CD and KED) to 200 broilers of 36, 42, or 43 days old on 3 experimental days. On days 2 and 3 an additional method was added, in which the bird’s head was extended at a ~90° angle after the application of the KED (KED+). Our study indicated brain stem death occurred sooner when birds were euthanized with CD compared to KED or KED+; all reflex durations were sustained for longer in the KED and KED+ birds.

**Abstract:**

The aim was to assess the onset of brain stem death for two euthanasia methods—manual cervical dislocation (CD) versus the Koechner Euthanizing Device (KED). Over three days broilers of 36 (n = 60), 42 (n = 80), or 43 days old (n = 60) were euthanized. On days 2 and 3, a treatment was added in which the bird’s head was extended at a ~90° angle after application of the KED (KED+). On those days, gap size was recorded between the skull and atlas vertebra by 1-cm increments. The onset of brain death was assessed by recording the nictitating membrane reflex, gasping reflex and musculoskeletal movements (sec). Additionally, skin damage and blood loss were recorded (y/n). On all days, CD resulted in quicker loss of reflexes and movements compared to KED or KED+. Reflexes returned in 0–15% of CD birds, 50–55% of KED birds, and 40–60% of KED+ birds, possibly regaining consciousness. Skin damage occurred in 0% of CD birds, 68–95% of KED birds, and 85–95% of KED+ birds. On day 2 (*p* = 0.065) and 3 (*p* = 0.008), KED birds had or tended to have a narrower skull-to-atlas gap compared to CD and KED+ birds. Based on our results, CD would be the recommended method for broilers.

## 1. Introduction

Producers and veterinarians euthanize poultry on-farm for multiple reasons, yet most often because a bird is ill or injured [1] and unable to access food and water. From an animal welfare point of view, euthanasia should result in quick loss of consciousness, with minimal pain or discomfort. From an operator’s point of view, the method should be easy, safe, and mobile. Due to the vast number of chickens—including broiler chickens—kept in livestock production (circa 60 billion [2]), on-farm euthanasia occurs daily. Especially for heavy birds, this can be challenging for the operator. A common method is to dislocate the head from the neck manually, resulting in extensive damage to the spinal cord and rupture of major blood vessels in the neck [3,4,5]. Although cervical dislocation is assumed to be humane, brain electrical activity can persist for up to 30 s following cervical dislocation methods. However, the birds are considered unconscious and incapable of perceiving pain in the cerebral cortex of the brain [6]. 

Manual cervical dislocation (CD) is dependent on the ability of the operator, which is susceptible to fatigue [1,7], and repeated success can depend on birds’ weight, size, and age [2,8]. Thus, for broilers approaching 3 kg or more, the application of cervical dislocation can be problematic. The strength required to hold a market-age broiler chicken by its legs or by the base of the wings and pull down its neck to cause dislocation can be physically demanding for most people. Furthermore, European legislation [9] was amended in 2013, restricting the number of birds allowed to be euthanized with manual cervical dislocation for each trained operator to 70 each day at a maximum live weight of 3 kg per bird (~7 weeks of age). Therefore, alternative methods need to be investigated for euthanasia, especially for large farms and older birds.

Mechanical cervical dislocation is similar to manual cervical dislocation in that it is aimed at separating the skull from the spinal column and thereby damaging the spinal cord and rupturing the vertebral and carotid arteries [1], and is performed with the help of a tool such as the Koechner Euthanizing Device (KED). Some studies have shown that mechanical cervical dislocation, either neck crushing or stretching, may not lead to immediate brain death in turkeys or chickens based on brain activity in anesthetized birds [6,8,10]. A recent study compared the KED with CD in broilers at 7, 21, and 35 days old and found that the KED method took longer to induce insensibility and brain death [11]. Yet, the device may be more effective than CD in heavier birds, as the strength required to dislocate the neck could be too great to result in rapid humane death. Therefore, further research is warranted. The aim of this study was to use induced reflexes to assess the ability of two euthanasia methods (CD and KED) to produce insensibility and brain death in broilers.

## 2. Materials and Methods 

This experiment was approved by the Institutional Animal Care and Use Committee (IACUC) of Virginia Tech (protocol 18-124) and followed SOP#10, “Euthanasia Methods Approved for Poultry”, approved by the IACUC at the U.S. National Poultry Research Center (USNPRC). For this experiment we euthanized three batches of fully-fed broilers on three non-consecutive experimental days over a period of three weeks. The cervical dislocation methods aimed to dislocate the atlas cervical vertebra from the base of the skull, resulting in a rapid death. If any bird had not died from the euthanasia treatment within 4 minutes, it would be immediately euthanized by vent-to-beak electrocution. An overview of observers and operators per experimental day is shown in Table 1.

On day 1, 80 male (Ross 708) broilers originating from an unrelated study on poultry nutrition were utilized. The birds were euthanized at 42 days old (mean ± SD body weights: 2.65 ± 0.088 kg) by either manual cervical dislocation (CD) or with the Koechner Euthanizing Device (KED-C, Clear View Enterprises LLC, USA). CD was performed by a farm manager with 30+ years of experience (operator A, Table 1). The farm manager held the broiler’s legs with one hand and supported the body on his upper leg. He then grasped the head behind the skull with two fingers, quickly and firmly pulling the head down and against the knuckle of the first finger to stretch the neck and separate the skull from the atlas vertebra. Immediately after, the bird was placed in a metal killing cone (which was placed on a wall at an 180° angle, thus completely vertical) to facilitate observation of induced reflexes. The KED euthanasia was performed by a newly trained researcher (operator B, Table 1), following manufacturer recommendations [12]. A bird was placed on the floor in a prone/sternal position by another researcher with one or two legs restrained. The KED was gently placed at the base of the skull with the twin blades ventral and the single blade dorsal. The jaws were closed slowly until the blades were in light contact with the bird. Then the handles were quickly brought together, after which the bird was placed in a metal cone to observe reflexes. Brain stem death was assessed by observing the induced nictitating membrane (third eye lid) reflex and the gasping reflex, as described previously [8,13]. Time was recorded from the start of euthanasia until cessation of musculoskeletal movements. Although we aimed to immediately assess responses after euthanasia, the setup practically resulted in the first assessment approximately 8 s after application of either euthanasia method. We recorded the presence or absence of the nictitating membrane reflex—a brain stem reflex that is a primary indicator of brain death [13]. The nictitating membrane reflex was assessed by repeatedly and continuously touching the medial canthus of one eye, with birds being considered to maintain brain stem functionality if the third eyelid moved over the cornea. If needed, the eye lids were gently opened to assess this reflex. The second measure, the gasping reflex, was considered a brain stem reflex controlled by the cranial nerves [14]. The gasping reflex was assessed by visually observing whether birds showed opening and closing movements with their mouth. We did not record thoracic respiratory movements. Furthermore, the time until cessation of musculoskeletal movement (including vent, legs, and tail) was interpreted as the onset of brain death [15]. Finally, we recorded the presence of torn skin in the neck region (presence/absence). Two observers performed all measurements, alternating between the 2 methods every 5 to 7 birds (Table 1).

On day 2 and 3, broilers (Ross 708) from the same flock (a university production class project) were euthanized at 36 days old, and—a week later—at 43 days old (n = 60 per experimental day, 50:50 male:female, mean body weights at day 36: 2.13 kg). Besides the application of CD and KED, a treatment was added in which each bird’s head was extended at a ~90° angle after application of the KED (KED+). The additional head extension was aimed to cause more damage in the neck, potentially causing a quicker death. Essentially we theorized the KED+ as a 2-phase euthanasia method—first to separate atlas from skull by application of the KED, and second, to cause major damage to arteries by extending and rotating the head.

On day 2, CD, KED, and KED+ were performed by a single researcher with 38+ years of experience (operator C, Table 1). On day 3, a trained graduate student with 4+ years of experience (operator D, Table 1) performed all three methods in addition to the researcher from day 2. Broilers were placed into plastic cones headfirst at an approximate 45° angle from vertical. With the head extending from the end of the cone, each method was applied to 3 male and then 3 female broilers (differentiated by comb development and body size). Two observers (Table 1) evaluated the nictitating membrane reflex, gasping reflex, and presence of musculoskeletal movements at 15 s intervals, with the first assessment 15 s after euthanasia. This approach allowed us to record whether reflexes returned after absence (yes/no). Observations continued until no movements occurred for 60 s. The presence of torn skin and/or bleeding from the head–neck region or the mouth were then noted (present/absent). Furthermore, the size of the separation gap between the skull and the atlas cervical vertebra was recorded in 1 cm increments to the nearest cm (skull-to-atlas gap) by placing fingers in the gap, with the width of a finger approximately 1 cm. First euthanasia attempts were successful for all birds.

Reflex and movement residuals approached normal distribution based on the visual assessment of quantile-quantile (QQ) plots, with the exception of gasping durations on day 2. Additionally, on day 2 and 3, the skull-to-atlas gap residuals were normally distributed. Normally distributed data were analyzed in SAS 9.4 (SAS Institute, Cary, NC, USA) with mixed models. All dependent variables were scored by 2 observers on each experimental day, thus “observer” was included as a random factor in the models and “method” as an independent variable. Although “observer” did not have a significant impact on most output variables (univariate *p*-values between 0.299 and 0.967 for most, with *p* = 0.084 and *p* < 0.001 for 2 output variables on 2 experimental days), it was maintained as a random factor to truly represent study design and to be included with the calculation of model estimates.

Residuals from gasping reflex durations (day 2), returning reflexes, and presence of blood loss and torn skin were not normally distributed, thus data were analyzed with the Kruskal–Wallis Chi-square test, including a Bonferroni correction for pairwise comparisons. Results are presented as Least Square Means ± SEM or frequencies. 

## 3. Results

**Figure 1:**
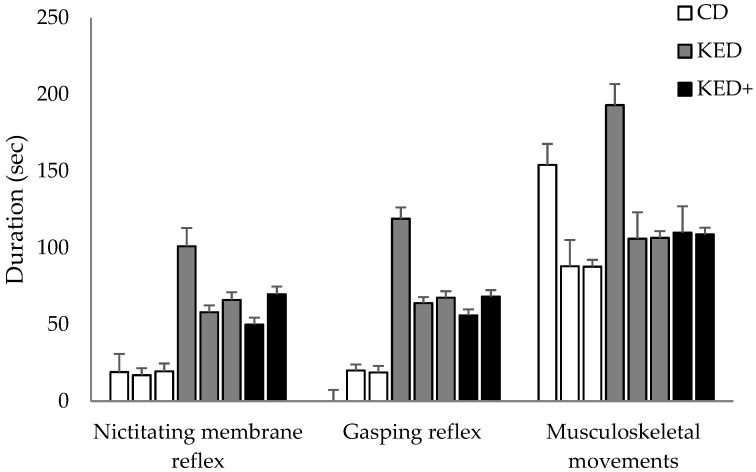
Durations for the nictitating membrane reflex (sec; LS Means ± SEM), gasping reflex, and musculoskeletal movements from three experimental days per euthanasia method (manual cervical dislocation, CD; Koechner Euthanizing Device, KED; Koechner device with additional head extension, KED+). Within “euthanasia method” each bar represents experimental day 1, 2, and 3, respectively. N = 80 for day 1, and 60 for day 2 and 3. N = 62 for gasping reflex on day 1.

Generally, both reflexes and musculoskeletal movements endured longer in KED and/or KED+ birds compared to CD birds on all experimental days. Durations of the nictitating membrane reflex were longer for the KED birds on experimental day 1 (*p* < 0.0001), and for KED and KED+ birds on day 2 and day 3 compared to CD birds (post-hoc *p* < 0.0001 for all pairwise comparisons; Figure 1). Similarly, gasping reflex durations were longer for KED birds compared to CD birds on day 1 (*p* < 0.0001), and KED and KED+ birds compared to CD birds on day 2 and 3 (Bonferroni-corrected *p* < 0.001 for all pairwise comparisons; Figure 1). Musculoskeletal movements endured longer in KED birds compared to CD birds on day 1 (*p* = 0.0002) and tended to be longer for KED+ birds compared to CD birds on day 2 (pairwise post-hoc *p* = 0.073). On day 3, movements endured longer in both KED (*p* = 0.0095) and KED+ birds (*p* = 0.003) compared to CD birds (Figure 1).

Returning reflexes (either nictitating membrane reflex or gasping reflex) were more common for KED and KED+ birds compared to CD birds (Bonferroni-corrected pairwise *p* = 0.008 and *p* = 0.092 for day 2 and *p* = 0.059 and *p* = 0.011 for day 3, respectively; Table 2). Skin damage was more frequent in KED birds compared to CD birds on day 1 (*p* < 0.0001), with the KED resulting in bleeding neck wounds in 24/40 birds (60%), and neck wounds without external bleeding in an additional 3/40 birds (8%). No torn skin was observed in CD birds on day 1. Both skin damage and blood loss were more common in KED and KED+ birds compared to CD birds on day 2 and day 3 (Bonferroni-corrected *p* < 0.001 for all pairwise comparisons; Table 2). 

The skull-to-atlas gap differed between methods on day 3 (*p* = 0.0077), with KED birds showing smaller gaps than KED+ birds (post-hoc *p* = 0.038), KED birds showing smaller gaps than CD birds (post-hoc *p* = 0.010), and no difference between KED+ and CD (*p* = 0.88; Table 2). The gap tended to differ on day 2, with a smaller gap in KED birds compared to CD birds (*p* = 0.065; Table 2). 

## 4. Discussion

This study showed a significant difference between KED, KED+, and CD euthanasia for duration of reflexes, onset of brain death, skin damage, and external blood loss, with KED and KED+ birds showing higher values for all measures.

A limitation of the euthanasia application on experimental day 1 was the difference in experience of the two people performing the euthanasia methods, with the KED operator newly trained versus 30+ years of experience for the CD operator. Some studies have identified training to have an impact on euthanasia success for a novel cervical dislocation method [2], or a similar method using pliers [8]. Anecdotally, the newly trained operator from day 1 did deem the KED method to be easier, requiring less skill and strength compared to the CD method, in which they were experienced. Yet, this argument does not account for differences in CD movement durations, as this was performed by an experienced operator. Experience with the KED was not the sole difference between experimental days, as birds were euthanized before placement in the cone (day 1), rather than after placement into the cone (day 2 and 3), the cones were placed differently (180 vs 45° angle) and observations were done continuously (roughly every second, day 1) rather than every 15 s (day 2 and 3). Despite differences in operator experience between day 1 vs. day 2 and 3, all euthanasia on day 2 and 3 was conducted by an experienced operator and a significant number of failures were still observed for the KED and KED+ methods, showing that the CD method consistently scores better in a variety of applications.

Brain death was estimated based on cessation of musculoskeletal movements and induced reflexes. Previous research showed that brain death was indicated by electroencephalogram silence in hens [16] and with the use of accelerometers, cessation of activity occurred at or about the same time as brain death in broilers [15]. The nictitating membrane reflex can persist for a short time (44 s after sodium pentobarbital intravenous euthanasia in [13]), and thus appears to be the most conservative measure to confirm brain death. Yet from an animal welfare standpoint, latency to brain death is not as important as latency to unconsciousness, which occurs under general anesthesia with the nictitating membrane reflex present.

The reported durations of the nictitating membrane reflex were probably overstated for CD birds on all experimental days, as for a majority of CD birds the nictitating membrane reflex was absent upon the first observation (approximately 8 s after the bird was placed in the cone on day 1, and at 15 s on day 2 and 3). It could be that in those birds the reflex ended prior to first assessment. Strong clonic or tonic convulsions and musculoskeletal movements also made initial observations of the nictitating membrane reflex difficult in some birds, potentially skewing durations upwards, with birds actually losing the reflex earlier than recorded.

Reflexes and movements remained longer with the KED and KED+ method compared to CD. In line with previous findings on 21- and 35-day-old broilers, the nictitating reflex and convulsions remained longer in KED compared to CD applications, indicating a longer time until brain death [11]. Our recorded duration of the gasping reflex for KED (119 s on day 1) was very similar to the 109 s that was observed in turkey hens euthanized with a Burdizzo (spinal cord crushing/cervical dislocation [8]). Cessation of activity occurred at about the same time as brain death, shown with the help of accelerometers [15]. Our results also showed that physical movement remained longer in KED birds than in CD birds, thus the onset of brain death was delayed.

We observed that reflexes returned in 40–60% of KED and KED+ birds, which could be due to a lack of brain stem damage or insufficient reduction of blood supply, as shown in rats [17]. The KED, and other plier-type devices, cause limited localized trauma rather than greater (and wider) damage due to the stretching of the neck and spinal cord during CD [1], which includes stretching the neck and spinal cord [1]. Earlier findings with other pliers in anesthetized hens showed that without severing the spinal cord, blood flow continued to the brain, increasing the time to loss of consciousness [4]. In line with our findings, no return of reflexes in CD birds was observed [8]. Gasping can occur in both conscious and unconscious animals, depending on the definition [3]. The gasping reflex is associated with “respiratory autoresuscitation”, and occurs when normal breathing ceases, in a final attempt to ventilate the lungs with oxygenated air. If animals are deemed conscious it may indicate breathlessness [18], which can be distressing. It could be possible that KED euthanasia resulted in crushing of the esophagus or trachea, but this was not confirmed in the current study. Similar to a previous study [19], we cannot confirm whether these birds were conscious but interpreted the gasping behavior as a brain stem reflex. 

As a third variable, we recorded the latency to last musculoskeletal movement. We interpreted the cessation of musculoskeletal movement to indicate brain death [15]. Clinical death occurs with cardiac arrest [3]. Yet, previous research shows that death occurs in phases, starting with suppression of brain activity, loss of responses to external stimuli, onset of convulsions, cessation of convulsions, the onset of brain death, and finally cardiac arrest (reviewed by [14]), although not all phases will occur for every euthanasia method. In our current study, we considered brain death to be more relevant than cardiac arrest. Our findings showed that, in line with a study on broilers [11], brain death was quicker in CD birds compared to KED birds.

Besides longer durations for reflexes and musculoskeletal movements, skin damage with external bleeding occurred frequently in KED birds. In CD birds, no skin damage or external bleeding was observed. Laceration of the skin with external blood loss can be a biosecurity hazard for both animals and people [20]. Furthermore, external damage could be deemed visually unpleasant to the operator. With mechanical cervical dislocation intended to serve as an alternative to manual dislocation, we included an additional potential application with KED, in which the head was extended at a ~90° angle after closing the pliers, with the aim to increase local trauma by inducing additional separation between the skull and atlas. Although we did record a similar skull-to-atlas gap in KED+ birds compared to CD birds, suggesting the trauma caused by KED+ was similar to CD, the reflexes and musculoskeletal movements endured, showing no significantly reduced latency to brain death. Thus, other alternatives should be considered that could result in brain death more quickly for KED or KED+ in the current study. For instance, the application of the Turkey Euthanasia Device, a non-penetrating captive bolt device, on broilers resulted in a lack of nictitating membrane response within 2 s, and the onset of death within 2 minutes (with death indicated by lack of respiration or heartbeat [21]). Thus, a captive bolt method may lead to quicker brain death than CD and KED in our current study.

The manufacturer states that the KED can be used for birds up to ~13 kg (KED-C). We found that the KED-C consistently scored worse compared to CD, both for younger, lighter birds, as for older, heavier birds.

Based on the longer duration of induced reflexes and musculoskeletal movement, and the return of reflexes in approximately half the birds, the KED (and KED+ approach) appears to delay the onset of brain death compared to CD. In line with this conclusion, plier-type devices are not permitted in the new EU legislation [9], or in UK RSPCA guidelines for broiler chickens [22]. 

## 5. Conclusions

We compared time to loss of induced reflexes and the onset of brain death in 36-, 42- and 43-day-old broilers when euthanized by manual cervical dislocation or mechanical cervical dislocation using the Koechner Euthanizing Device. In our experiment, reflexes were lost sooner, brain death occurred faster, internal damage was greater, and external damage was absent after application of manual cervical dislocation compared to the Koechner methods. Although time to loss of consciousness was not estimated, manual cervical dislocation would be the recommended method for broilers.

## Figures and Tables

**Table 1:** Number of birds recorded by four observers in three batches of broilers euthanized by manual cervical dislocation (CD), Koechner Euthanizing Device (KED), and KED with additional head extension (KED+). Experimental days spanned over a time period of three weeks.

		Day (Batch) ^1^							
		1		2			3		
		CD	KED	CD	KED	KED+	CD	KED	KED+
**Observer** ^2^	1	16	24	9	10	10			
	2	23	17						
	3			11	10	10	13	13	13
	4						7	7	7
**Operator** ^3^		A	B	C			C/D		

^1^ Empty cells indicate no observations by that specific observer. ^2^ Person recording output variables (e.g., reflexes, movements). ^3^ Person(s) performing euthanasia (can be an observer too).

**Table 2:** Number of birds in which reflexes returned after scored absent per euthanasia method (manual cervical dislocation, CD; Koechner Euthanizing Device, KED; Koechner device with additional head extension, KED+), number of birds with skin damage and bleeding, and gap size (LS Means ± SEM) between skull and atlas vertebra for experimental day 2 and 3.

Method	Returning Reflexes				Torn Skin				External Blood Loss				Skull-to-Atlas Gap	
	Day 2		Day 3		Day 2		Day 3		Day 2		Day 3		Day 2	Day 3
	N	%	N	%	N	%	N	%	N	%	N	%	cm	
CD	2	10	3	15	0	0	0	0	0	0	0	0	1.97 ± 0.20	1.63 ± 0.15
KED	11	55	10	50	15	75	19	95	15	75	17	85	1.28 ± 0.22	1.00 ± 0.15
KED+	8	40	12	60	17	85	19	95	17	85	18	90	1.81 ± 0.20	1.53 ± 0.15
